# Role of induced-strain and interlayer coupling in contact resistance of VS_2_–BGaX_2_ (X = S, Se) van der Waals heterostructures

**DOI:** 10.1039/d5na00356c

**Published:** 2025-07-28

**Authors:** Umair Khan, Basit Ali, Tahani A. Alrebdi, M. Bilal, M. Shafiq, M. Idrees, Bin Amin

**Affiliations:** a Department of Physics, Abbottabad University of Science & Technology Abbottabad 22010 Pakistan binukhn@gmail.com +92-333-943-665; b Department of Physics, College of Science, Princess Nourah Bint Abdulrahman University P. O. Box 84428 Riyadh 11671 Saudi Arabia; c School of Chemistry and Chemical Engineering, Shandong University Jinan 250100 P. R. China

## Abstract

Using Density Functional Theory (DFT) calculations, we explored the electronic band structure and contact type (Schottky and Ohmic) at the interface of VS_2_–BGaX_2_ (X = S, Se) metal–semiconductor (MS) van der Waals heterostructures (vdWHs). The thermal and dynamical stabilities of the investigated systems were systematically validated using energy–strain analysis, *ab initio* molecular dynamics (AIMD) simulations, as well as binding energy and phonon spectrum calculations. After analyzing the band structure, VS_2_–BGaX_2_ (X = S, Se) MS vdWHs metallic behavior with type-III band alignment is revealed. A p-type Schottky (Ohmic) contact in VS_2_–BGaS_2_ (VS_2_–BGaSe_2_) MS vdWHs with decreasing (increasing) tunneling probabilities (current) shows its potential uses in phototransistors, photodetectors and high-speed nanoelectronic devices. Additionally, the work function (*ϕ*), electrostatic potential and charge density difference are also investigated to gain detailed insights into the work function variations and charge transfer between layers during the fabrication of VS_2_–BGaX_2_ (X = S, Se) MS vdWHs. At equilibrium interlayer distance, strong interlayer coupling due to the vdW interactions is further confirmed *via* Bader charge analysis, showing that the electrons are transferred from BGaS_2_(VS_2_) to the VS_2_(BGaS_2_) layer in VS_2_–BGaS_2_ (VS_2_–BGaSe_2_) MS vdWHs. These calculations give a new strategy for experimentalists to design advanced high-speed nanoelectronic devices based on VS_2_–BGaX_2_ (X = S, Se) MS vdWHs.

## Abstract 2

Using Density Functional Theory (DFT) calculations, we explored the electronic band structure and contact type (Schottky and Ohmic) at the interface of VS_2_–BGaX_2_ (X = S, Se) metal–semiconductor (MS) van der Waals heterostructures (vdWHs).

## Graphical abstract

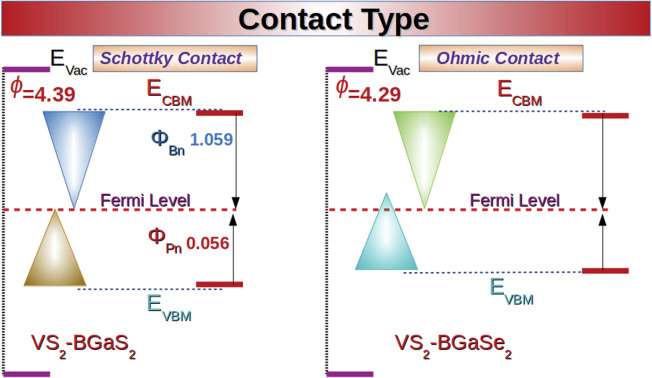

## 1. Introduction

Practical applications of semiconductors require direct contact with the metal electrode in the form of a metal–semiconductor (MS) contact to achieve efficient carrier injection for high-performance nanoelectronic devices.^1–3^ In the MS contact, although the Schottky barrier (SB) allows carriers to pass between the metal and semiconductor,^4^ its height (SBH) poses critical problems to the charge carriers’ injection capability in devices.^5^ The formation of proficient nanodevices depends on the modulation of contact resistance (Schottky or Ohmic) at the MS interface.^6^

Heavy doping is used in conventional bulk materials to achieve low contact resistances, but this technique is not easy in silicon-based FETs (using bulk metal electrodes).^7–10^ The ultrathin nature of 2D materials also does not allow heavy doping in the contact area, as it would vary their structure and other properties. Fermi level pinning (FLP) at the interface of the 3D metal and 2D semiconductor also limits the drain current and charge injection efficiency.^11–15^ To find a metal with a suitable work function that also exhibits high conductivity and chemical and thermal stability to obtain a Schottky or Ohmic contact with semiconductors is also very difficult.^16^ Due to the low dimension scale of devices (less than 10 nm), tuning the SBH at the MS junctions *via* an external electric field also poses substantial challenges.^17^

Stacking of two-dimensional (2D) materials to form a van der Waals heterostructure (vdWH) has emerged as an effective strategy to enhance material properties and uncover novel physical phenomena. This approach is widely used in design and development of next-generation nanoelectronic devices with tailored functionalities, which often depend on the type of band alignment at the interface. Typically, the type of band alignment is categorized into three types on the basis of the relative positions of the conduction band (CB) and valence band (VB) edges of the constituent layers. In type-I (straddling gap) band alignment the contributions of the CB and VB originate from same layer, effectively confining holes and electrons within one material. While in type-II (staggered gap) alignment the CB and VB are contributed by distinct layers, facilitating efficient charge separation across the interface, and in type-III (broken gap) band alignment either the CB or VB of one layer crosses the Fermi level during the contact, resulting in a band overlap at the Fermi level which can facilitate tunneling behavior and is particularly useful in designing photodetectors, tunneling transistors, and other advanced electronic devices. In this context, understanding the band alignment at the interface of metal–semiconductor heterostructures is crucial, significantly influencing charge transfer and contact resistance.^18,19^

Stacking of metals with semiconductors in the form of 2D vdWHs also prevents the development of localized electronic states and allows the Fermi level freely to align with the semiconductor’s valence or conduction bands forming a p-/n-type Schottky or Ohmic contact at the interface.^20,21^ This reduces the FLP and potential scattering, thereby enhancing device performance.^22,23^ Tuning of the SBH by varying the interfacial distance in vdWHs has been previously reported in ref. 24 and 25, offering a promising approach for SBH modulation and optimizing charge injection and carrier transport. Although, in the 2D family, transition metal dichalcogenides (TMDCs) are widely used in MS contacts,^26–28^ members of the same family with metallic behaviour have also received significant attention for a variety of nanodevice applications.^29–33^ The work functions of TMDCs align well with several other semiconducting materials, making them suitable for contact electrodes in FETs, lowering the SBH and enhancing the device efficiency.^6^ The unique and complicated electronic structure of VS_2_ has inspired us to explore this layered compound as a promising functional material. The 2D electron–electron correlations among the V atoms are expected to induce more intricate planar electric transport properties.^34,35^ In the same family of 2D materials, Janus TMDCs (MoSSe)^36,37^ have opened a pathway for the designing of novel Janus-materials.^38–40^ Structural and mechanical stability, optical and ferro-piezoelectric properties of Janus BGaS_2_ and BGaSe_2_ have recently been explored *via* first-principles calculations.^41^ Both BGaS_2_ and BGaSe_2_ are semiconductors with indirect bandgaps of 2.13 eV and 1.63 eV, respectively.

In this work, using first principles calculations, vdWHs in the form of MS contacts of VS_2_ with Janus BGaS_2_ and BGaSe_2_ layers are modelled, and their contact properties are explored. The experimentally accessible lattice mismatch between the constituent layers ensures that the VS_2_–BGaX_2_ (X = S, Se) vdW interfaces remain energetically viable while preserving their intrinsic properties. These findings demonstrate that the VS_2_–BGaX_2_ (X = S, Se) vdW interfaces exhibit an adjustable SBH, and the contact type can be switched from p- to n-type and from Schottky to Ohmic. Therefore, due to their tunable barrier height, these vdW interfaces are highly suitable for enhancing the carrier injection capability with promising applications in light-emitting diodes (LEDs) photodetectors and solar cells.

## 2. Computational details

Density Functional Theory (DFT) calculations were carried out using the PWSCF code^42^ with employing the Perdew–Burke–Ernzerhof (PBE) exchange–correlation functional.^43^ A plane-wave cutoff energy of 700 eV was adopted to ensure accurate total energy and forces evaluations. For the geometry optimization a Monkhorst–Pack *K*-point grid of 8 × 8 × 1 was used while a denser grid of 16 × 16 × 1 was applied for the self-consistent field. The convergence thresholds were set to 10^−3^ eV Å^−1^ and 10^−4^ eV, for force and energy respectively, ensuring reliable optimization of lattice parameters and atomic position. To eliminate spurious interactions between periodic images, a vacuum space of 25 Å was introduced along the out-of-plane (*z*-direction). All binding energy and structural relaxation calculations were carried out using the PBE functional combined with Grimme’s DFT-D2 dispersion corrections to accurately capture long-range van der Waals interactions.^44^

Using *ab initio* molecular dynamics (AIMD) simulations,^45^ the thermal stability was investigated *via* the Nosé thermostat algorithm at a temperature of 300 K, and the dynamical stability through phonon spectra using density functional perturbation theory (DFPT).^46^ The mechanical stability of these systems is confirmed from the elastic constants calculated *via* the energy–strain approach.^47^ The interlayer distance and binding energies for all stacking configurations of VS_2_–BGaX_2_ (X = S, Se) are calculated *via*:^48^*E*_b_ = *E*_(vdWH)_ − *E*_(semiconducting-layer)_ − *E*_(metallic-layer)_.

Using the Schottky–Mott rule,^49^ the heights of the n-type (*Φ*_Bn_ = *E*_CBM_ − *E*_F_) and p-type (*Φ*_Bp_ = *E*_F_ − *E*_VBM_) Schottky contacts are calculated (*E*_CBM_ and *E*_VBM_ are the energies of the band edges of the semiconducting material, *E*_F_ is the Fermi level of the vdWH). To confirm the charge transfer and type of contact, band bending is also calculated^50^*via*: Δ*ϕ* = *ϕ*_VS_2__ − *ϕ*_BGaX_2__, where *ϕ*_VS_2__ and *ϕ*_BGaX_2__ denote the work function of the corresponding metallic (VS_2_) and semiconducting (BGaX_2_) layers, respectively in the MS vdWHs. The tunneling barrier probability *via*:^49^
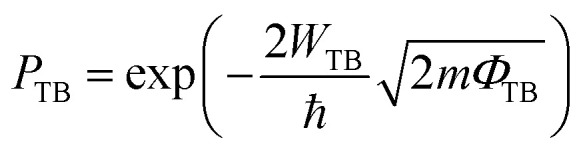
 (*m* is the mass of a free electron, and ℏ is the reduced Planck constant) is expressed in terms of the comprehensive factor: *C* = (*W*_TB_)^2^*Φ*_TB_.

## 3. Results and discussion

**Fig. 1:**
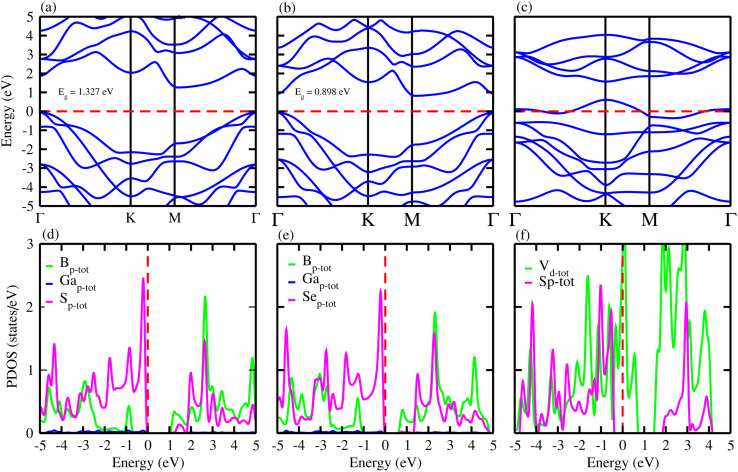
Electronic band structure (first row) and density of states (second row) of: (a and d) BGaS_2_, (b and e) BGaSe_2_, and (c and f) VS_2_ (see text for details).

BGaX_2_ (X = S, Se) in hexagonal close-packing has a four-layer atomic structure, composed of BX and GaX layers. Each B atom is covalently bonded to two X atoms and one Ga atom (X–B–Ga–X), leading to a trigonal prismatic geometry (space group no. 187 (*P*6̄*m*2)). Substituting B with a Ga atom disrupts the out-of-plane symmetry from *D*_3h_ to *C*_3v_, which significantly affects the electronic structure and optical properties of these materials.^41,51^ BGaS_2_ (Fig. 1(a)) and BGaSe_2_ (Fig. 1(b)) are indirect bandgap (*Γ*–*M*) semiconductors, while VS_2_ is metal (Fig. 1(c)). In the case of the BGaS_2_ (BGaSe_2_) layers, the valence band near the Fermi level is mainly due to B-p and Ga-p orbitals, while the conduction band is due to B-p and S(Se)-p orbitals confirming the semiconducting behavior, as shown in Fig. 1(d) and (e). In the VS_2_ layer (see Fig. 1(f)), V-d and S-p orbitals cross the Fermi level, confirming the metallic behaviour. The lattice constants, bond lengths, bond angles along with the band gap energies of BGaS_2_ and BGaSe_2_ in Table 1 are in good agreement with previous work.^41^

**Table 1:** Lattice constant (*a* in Å), bond length (B–Ga, B–X, Ga–X (X = S, Se) in Å), layer thickness (S–Se in Å) between chalcogen atoms, bond angles, the energy band-gap (*E*_g_ in eV) and the elastic constants (C11, C12, C66) of BGaS_2_, BGaSe_2_, VS_2_–BGaS_2_ and VS_2_–BGaSe_2_ MS vdWHs

Material	*a*	B–Ga	B–X	Ga–X	S–Se	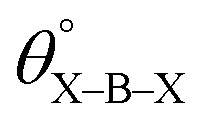	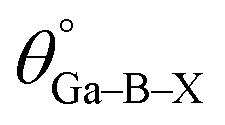	*E*_g_	*C*_11_	*C*_12_	*C*_66_
BGaS_2_	3.29	2.09	2.06	2.26	4.11	105.97	112.77	1.33	58.67	14.43	22.12
BGaSe_2_	3.46	2.07	2.19	2.39	4.38	104.51	114.06	0.91	51.22	10.25	20.48
VS_2_–BGaS_2_	3.23	2.08	2.03	2.25	4.15	105.51	113.18	1.327	88.36	28.67	29.84
VS_2_–BGaSe_2_	3.32	2.07	2.12	2.38	4.39	102.94	115.40	0.898	74.23	48.52	12.85

The carrier effective mass (*m*) is a crucial parameter in semiconductor physics, as it influences charge carrier mobility, electrical conductivity, and overall device performance. It depends on the curvature of the conduction and valence bands. A smaller effective mass leads to higher carrier mobility *via*: 
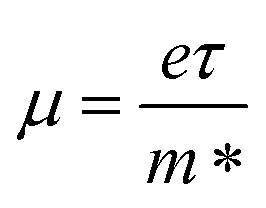
,^52^ making materials more suitable for high-speed electronic and optoelectronic applications. The carrier effective mass in BGaX_2_ (X = S, Se) layers is investigated *via*
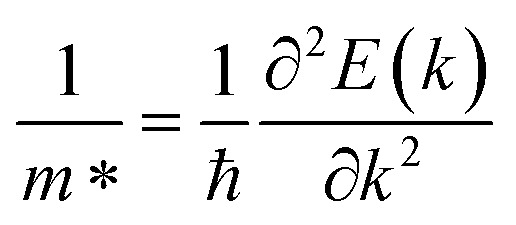
 by parabolic fitting of the CBM and VBM.^53,54^ The calculated effective mass of electrons (holes) in BGaS_2_ is 0.0722 (0.205) and in BGaSe_2_ is 0.091 (0.187).

**Fig. 2:**
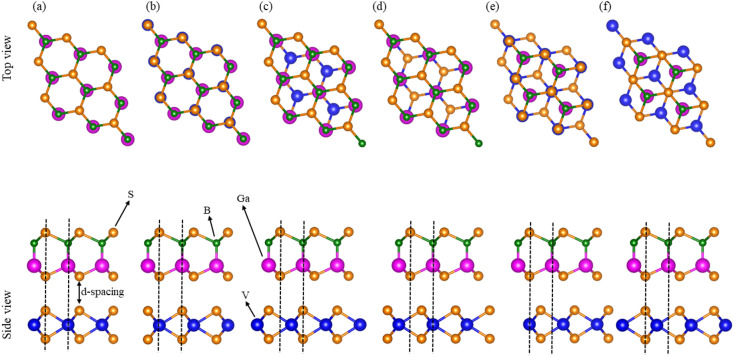
Geometrical structures of the VS_2_–BGaX_2_ (X = S, Se) van der Waals heterostructure in various stacking patterns (see text for details).

The lattice mismatch of VS_2_ and BGaS_2_ monolayers in the modelling of the VS_2_–BGaS_2_ vdWH is 3.5%. and in the case of the VS_2_–BGaSe_2_ vdWH it is 8.3%, which realizes experimental fabrication. Therefore, to understand the formation of the contact between the 2D metallic VS_2_ and semiconducting BGaX_2_ (X = S, Se) layers, we modelled six possible stackings ((a)–(f)) of VS_2_–BGaX_2_ vdWHs in a 1 × 1 unit cell of each layer conforming to the minor lattice mismatch (<10%),^55^ which does not substantially impact the fundamental properties of the constituent layers, see Fig. 2. In the case of (a), an X(B and Ga) atom of BGaX_2_ is placed on top of an S(V) atom of the VS_2_ layer. In (b), the X(B and Ga) atom of BGaX_2_ is settled on top of the V(S) atom of the VS_2_ layer. In (c), the B and Ga(X) atoms of BGaX_2_ are positioned on top of an S(hollow-site) of the VS_2_ layer. Similarly in (d), the B and Ga(X) atoms of BGaX_2_ are placed on top of a V(hollow-site) of the VS_2_ layer. In (e), the BGaX_2_ X(B and Ga) atoms are placed on top of a V(hollow-site) of the VS_2_ layer. In (f), the X(B and Ga) atoms of BGaS_2_ are placed on top of an S(hollow-site) of the hexagonal lattice site of the VS_2_ layer.

The calculated binding energy and the interlayer distance in Table 2 show the experimental fabrication of all possible stacking configurations. The most stable stacking configuration is the one with the most negative binding energy and the shortest interlayer distance, see Table 2. Among these six stacking configurations, the stacking (b) is found to be the most favorable for these MS vdWHs. The calculated binding energies (−0.24 to −0.33 eV) fall well within the range typical for vdWHs, confirming the non-covalent bonding between the interlayers, hence supporting the physical nature of the heterostructure interface without significant chemical reconstruction. During the modeling of VS_2_–BGaX_2_ MS vdWHs, strain is induced in the corresponding layers due to their lattice mismatch that significantly impacts the stability of the various stacking configurations of the MS vdWHs. Therefore, we re-optimized the lattice constant of the VS_2_–BGaX_2_ MS vdWHs, and the results are presented in Table 1.

**Table 2:** Binding energy (*E*_b_ in eV) and inter-layer distance (*d* in Å) of VS_2_–BGaS_2_ and VS_2_–BGaSe_2_ MS vdWHs

Material		(a)	(b)	(c)	(d)	(e)	(f)
VS_2_–BGaS_2_	*E*_b_ (eV)	−0.331	−0.333	−0.329	−0.331	−0.332	−0.328
	*d* (Å)	3.320	3.170	3.240	3.301	3.302	3.250
VS_2_–BGaSe_2_	*E*_b_ (eV)	−0.240	−0.242	−0.238	−0.239	−0.241	−0.240
	*d* (Å)	3.320	3.200	3.450	3.430	3.250	3.290

**Fig. 3:**
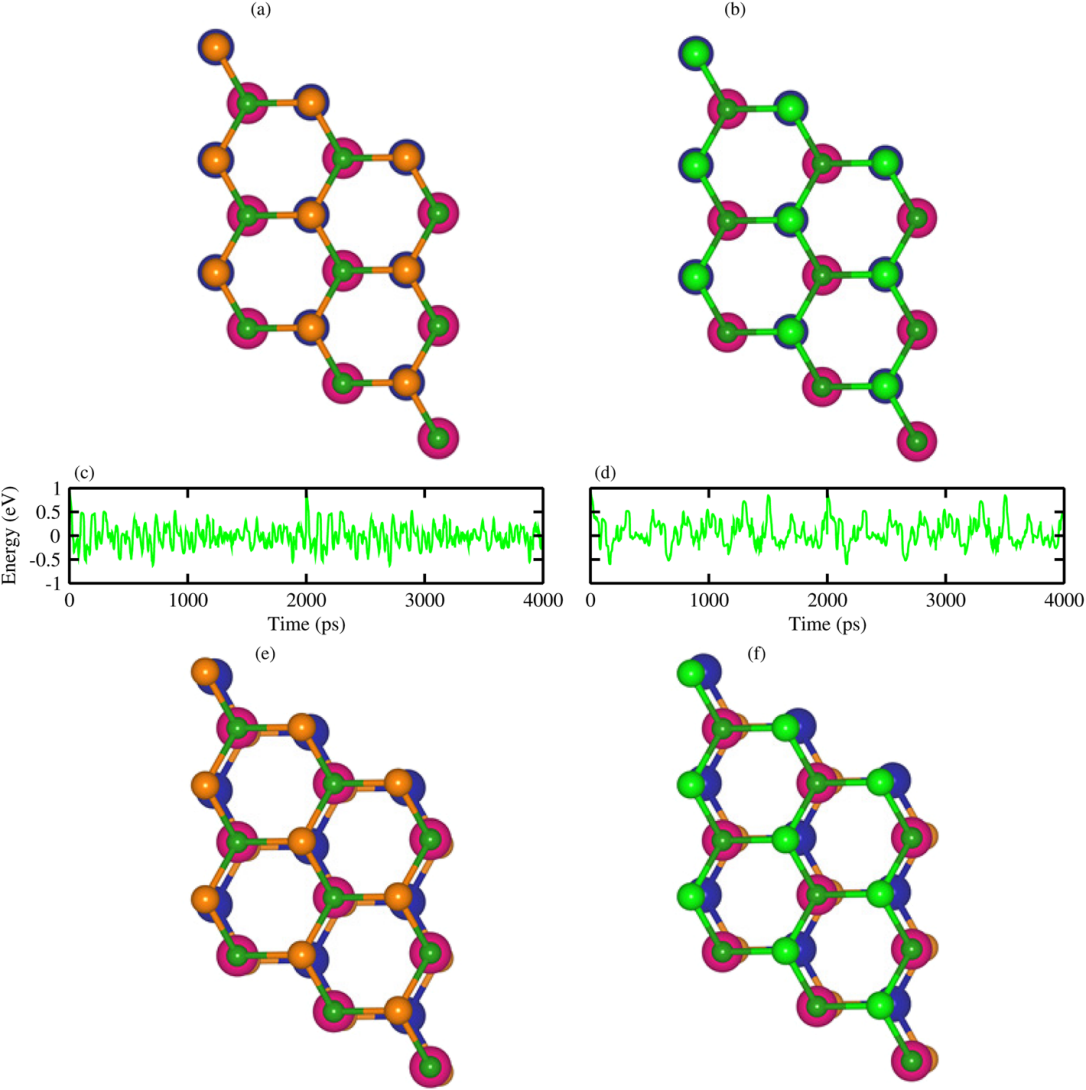
Geometrical structures before heating (first row), with fluctuating energy (second row) and after heating (third row) of (a) VS_2_–BGaS_2_ and (b) VS_2_–BGaSe_2_ (see text for details).

Using AIMD simulations, the geometrical structure is found to remain unchanged (no structural distortions are observed) after 4000 step simulations at room temperature (300 K) with nearly constant fluctuation energy, see Fig. 3. This confirms the thermal stability of VS_2_–BGaX_2_ MS vdWHs.^56^

**Fig. 4:**
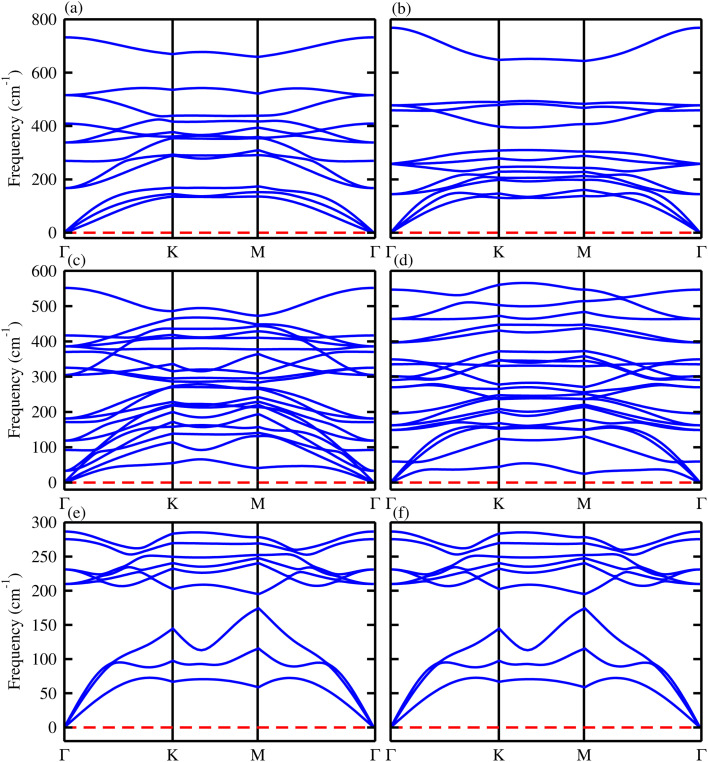
Phonon spectra of (a) BGaS_2_, (b) BGaSe_2_, (c) VS_2_–BGaS_2_, (d) VS_2_–BGaSe_2_ and (e and f) VS_2_ (see text for details).

In agreement with ref. 34, 35 and 41, the phonon dispersion curves of the 2D BGaS_2_, BGaSe_2_, and VS_2_ layers, along with their vdWHs (VS_2_–BGaS_2_ and VS_2_–BGaSe_2_) are calculated in the high-symmetry directions (*Γ*–*K*–*M*–*Γ*) of the BZ. The absence of imaginary frequencies confirm the dynamical stability of these systems. The BGaX_2_ (X = S, Se) layer contains four atoms per unit cell exhibiting a total number of 12 modes, see Fig. 4(a) and (b). The three lower frequency modes correspond to the in-plane longitudinal (LA), transverse (TA) and out-of-plane acoustic (ZA) modes. The remaining nine modes indicate the optical branches are well-separated, having a higher frequency of ∼800 cm^−1^. VS_2_ in Fig. 4(e) and (f) consists of three atoms per unit cell having a total number of nine modes. The highest optical phonon frequency is ∼300 cm^−1^, significantly lower than BGaX_2_ compounds. This suggests weaker bonding and stronger electron–phonon coupling, consistent with the VS_2_ metallic nature (metals often have strong interactions between electrons and phonons). In the case of VS_2_–BGaX_2_ MS vdWHs, there are a total of 21 modes of frequency, due to the seven atoms per unit cell see Fig. 3(c) and (d). Due to the weak vdW interactions, the 3 lower frequency modes are acoustical (LA, TA and ZA) and the remaining 18 modes are optical. The general shapes of the phonon dispersion for VS_2_–BGaX_2_ MS vdWHs resemble a simple superposition of the phonon dispersions of the corresponding layers (VS_2_, BGaX_2_). Due to the metallic nature of the VS_2_ monolayers and interlayer coupling with BGaX_2_, the frequency ranges become lower in vdWHs up to ∼500 cm^−1^. These investigations confirm that the structure of the VS_2_–BGaX_2_ MS vdWHs do not impulsively collapse, making these materials well suited for further tuning, thereby opening a pathway for feature field effect transistors, optoelectronics and nanoelectronic devices.^57,58^

The lattice mismatch introduced induced strain in BGaS_2_, BGaSe_2_ and VS_2_ layers may significantly alter the bond length. Therefore, elastic constants (*C*_*ij*_)^59,60^ of the vdWHs and corresponding layers are calculated. As shown in Table 1, *C*_11_ > 0, *C*_12_ > 0, *C*_11_ > ∣*C*_12_∣, satisfying the Born criteria^61,62^ for both vdWHs and their corresponding layers, which indicates high mechanical stability. The higher values of *C*_11_ in Table 1 (BGaS_2_ > BGaSe_2_ > VS_2_) suggest that BGaS_2_ is stiffer than BGaSe_2_ and VS_2_, hence confirming that BGaS_2_ is more resistant to deformation. In the case of VS_2_–BGaX_2_ (X = S, Se) MS vdWHs, additional stress fields are generated at the interface between the corresponding layers. These stress fields can introduce interfacial dipoles that significantly alter the elastic properties such as stiffness and shear resistance, making these material more anisotropic compared to the individual layers. For the vdWHs, the higher value of *C*_11_ compared to the corresponding layers indicates that the interaction between layers leads to enhanced in-plane stiffness, especially if the interlayer coupling is strong. The *C*_66_ parameter, derived from *C*_11_ and *C*_12_, is used to understand shear deformation within the basal plane, helping to assess the mechanical stability of the vdWHs. This parameter also provides insight into mechanisms of energy dissipation, such as interlayer sliding, which may occur under mechanical stress. These calculations suggest that the vdWHs are mechanically stronger due to interlayer vdW interactions and strain effects.

**Fig. 5:**
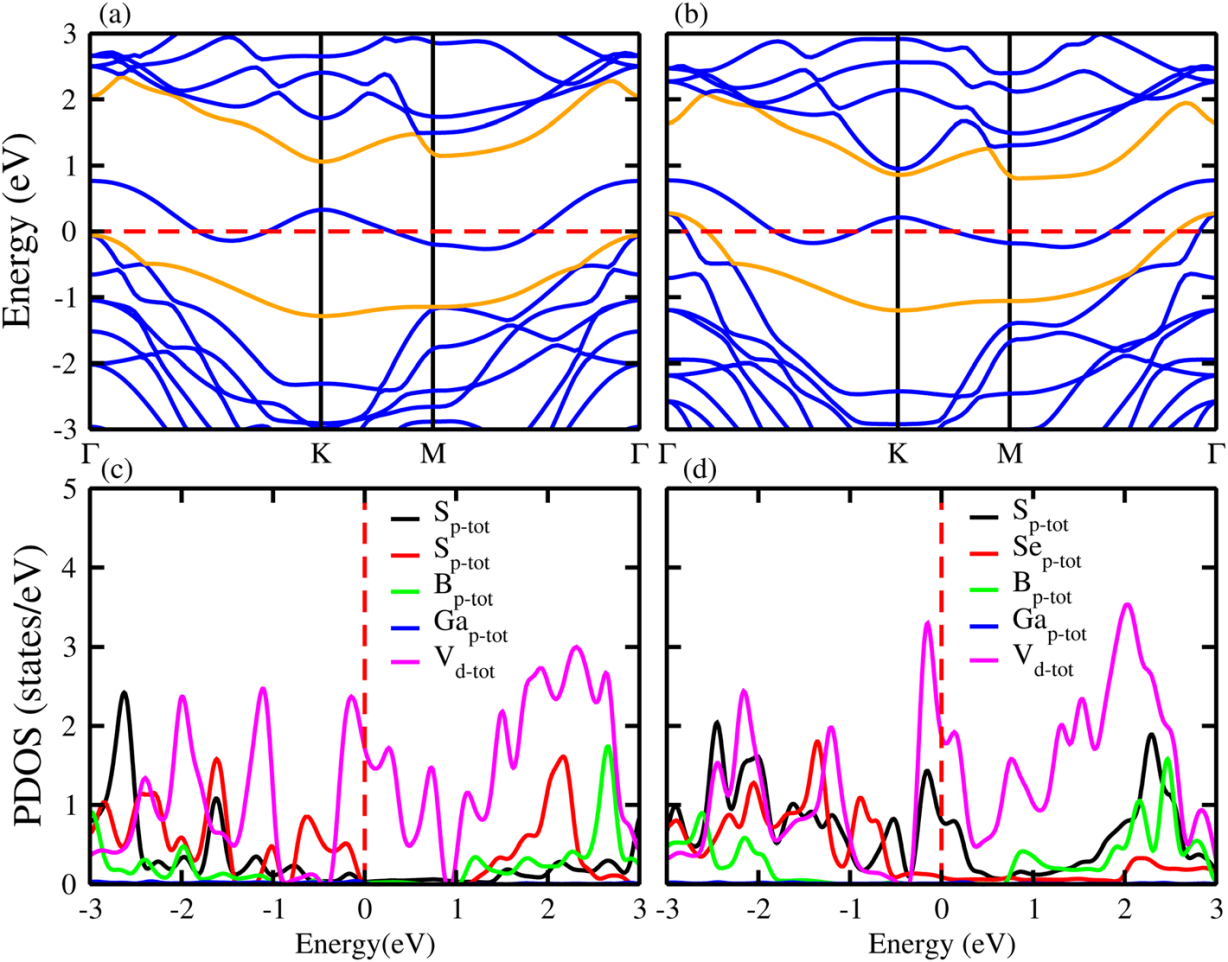
Electronic band structure (first row) and density of states (second row) of (a and c) VS_2_–BGaS_2_, (b and d) VS_2_–BGaSe_2_ vdWHs (see text for details).

The calculated electronic band structures of VS_2_–BGaS_2_ (Fig. 5(a)) and VS_2_–BGaSe_2_ (Fig. 5(b)) vdWHs display energy dispersion along *Γ*–*K*–*M*–*Γ*, where the blue (orange) lines represent the contribution of VS_2_ (BGaX_2_ (X = S, Se)) layers. Fig. 5(a) and (b) demonstrate that VS_2_–BGaX_2_ are metals with type-III band alignment.^63^ Due to the weak vdW forces at the interface, the band structures of VS_2_–BGaX_2_ vdWHs closely resemble the superposition of the band structures of their corresponding layers. Stacking in the form of VS_2_–BGaX_2_ MS vdWHs induces strain due to lattice mismatch between individual layers, leading to tuning of overall properties of the materials (the VBM of the BGaS_2_ layer shifts near to the Fermi level, while that of the BGaSe_2_ layer crosses the Fermi level). Additionally, the strong differences in electronegativity between the intrafacial atoms of the VS_2_ and BGaS_2_ (BGaSe_2_) layers encourage electron transfer across the interface, affecting the Schottky (Ohmic) contact resistance and also contributing to alteration of the type of contact (p-type and n-type) depending on the relative position of the metal’s Fermi level with respect to the semiconductor’s band edges (CBM and VBM) of MS vdWHs. Schottky contact occurs when there is a significant energy barrier for electron and hole transfer across the metal and semiconductors, while in the case of Ohmic contact the barrier for electron and hole injection is negligible or absent, allowing holes to move freely across the interface, which leads to low contact resistance and efficient carrier transport. In the case of n-type SBH, the conduction band of the semiconductors aligns closer to the Fermi level of the metal, facilitating electron flow from the metal to semiconductor. While in the case of p-type (SBH) contact, the valence band of the semiconducting layer is positioned closer to the metal Fermi level with holes as the majority carriers at the interface and allows hole transport from semiconductor to metal. Moreover, interfacial charge transfer creates a dipole layer at the interface, showing potential applications in FETs. The higher (lower) bandgap of BGaS_2_ (BGaSe_2_) implies that the VS_2_–BGaS_2_ (VS_2_–BGaS_2_) vdWHs exhibit Schottky (Ohmic) contact behavior. However, for future studies, the strong hybridization suggests that the barrier could be reduced, potentially leading to a transition toward Ohmic behavior under certain doping conditions or strain engineering. We have also calculated the band structure of other stacking configurations, see Fig. 3S in the supplementary information (SI).

The partial density of states (PDOS) of VS_2_–BGaX_2_ in Fig. 5 shows that the primary contribution to the Fermi level originates from the V-d and S-p orbitals of the VS_2_ layer (crossing the Fermi level). In the case of VS_2_–BGaS_2_, the S-p and Ga-p orbitals mainly contribute in the VBM, while the B-p orbitals dominate in the CBM, without crossing the Fermi level. In contrast, for VS_2_–BGaSe_2_, the Se-p orbitals cross the Fermi level, whereas the B-p and Ga-p orbitals do not significantly contribute to the band edges of VS_2_–BGaSe_2_, see Fig. 5(c) and (d) respectively. Both the electronic band structure and PDOS reveal that changing the chalcogen atom from S (BGaS_2_) to Se (BGaSe_2_), alters the band alignment, affecting the degree of band hybridization. In the case of VS_2_–BGaSe_2_ vdWH, Se leads to stronger hybridization with VS_2_ compared to S in VS_2_–BGaS_2_, potentially affecting interfacial charge transfer and contact resistance. These findings suggest that transition metal dichalcogenides (TMDs) like VS_2_ can serve as an excellent contact material for semiconductor-based heterostructures, enabling efficient charge transport in nanoelectronic and optoelectronic devices.

**Fig. 6:**
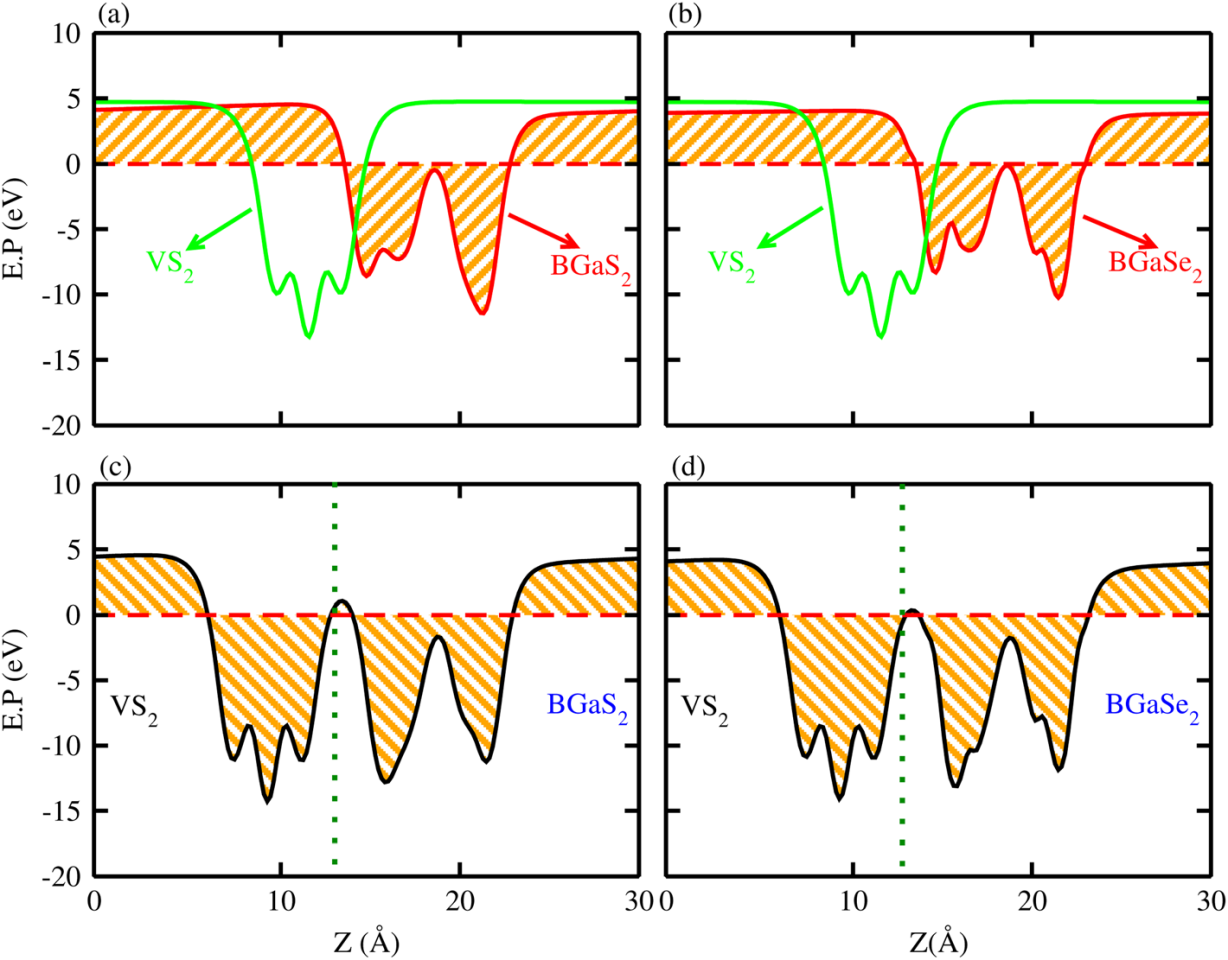
Electrostatic potential of (a) BGaS_2_, (b) BGaSe_2_, (c) VS_2_–BGaS_2_ and (d) VS_2_–BGaSe_2_ vdWHs. Where the green line is for the VS_2_ monolayer (see text for details).

The electrostatic potential (Δ*V*) plays a crucial role in understanding the charge distribution, band alignment, and interfacial interactions in vdWHs. Fig. 6 illustrates the variation in potential energy along the *Z*-direction for (a) BGaS_2_, (b) BGaSe_2_, (c) VS_2_–BGaS_2_ and (d) VS_2_–BGaSe_2_ vdWHs, while the green line is for the VS_2_ layer. Although, the Δ*V* of the VS_2_ layer exhibits a deeper potential then BGaX_2_ (X = S, Se) due to its strong electron localization and high carrier density. The stacking of VS_2_–BGaX_2_ (X = S, Se) MS vdWHs, due to strong electronegativity differences and charge redistribution at the interface, significantly modifies Δ*V*. Fig. 6(c), shows that the VS_2_ layer exhibits a deeper potential then BGaS_2_, confirming transportation of charge from BGaS_2_ to the VS_2_ layer in the VS_2_–BGaS_2_ MS vdWH. In contrast, for the VS_2_–BGaSe_2_ MS vdWH (Fig. 6(d)), charges are transferred from VS_2_ to the BaGaSe_2_ layer. This charge transport indicates a strong interlayer coupling arising from the vdW interfacial forces of VS_2_ and BGaX_2_ layers in the VS_2_–BGaX_2_ (X = S, Se) MS vdWHs.

In accordance with ref. 64 (for GaSe/graphene) and ref. 65 (for MoS_2_/graphene), charge transport induces a region of charge separation, which create an interfacial electric field within the heterostructure interface.^66^ This interfacial electric field increases the number of carriers, strengthens mobility, and tunes the work function (*ϕ*). The calculated *ϕ* of VS_2_ is 4.711 eV, BGaS_2_ is 4.099 eV and BGaSe_2_ is 3.867 eV. Although, due to the vdW interaction and potential alignment, the Fermi levels of the individual layers equilibrate upon stacking, resulting in band bending at the interfaces, having profound implications for charge carrier transport (discussed in detail below). The work function of these heterostructures becomes optimized and inferior to that of their respective monolayers. The *ϕ* values of VS_2_–BGaX_2_ (X = S, Se) vdWHs are 4.399 eV and 4.293 eV, respectively, which are lower than that of their corresponding layers facilitating efficient charge transfer. These materials show potential for device applications due to provision of an optimal threshold voltage that enhances carrier injection control, and allows for further tuning. The Δ*V* across the interface of VS_2_–BGaX_2_ (X = S, Se) vdWHs ranges from 0.143–0.244 eV. The results, along with the tunable work function and improved carrier mobility, indicate that these vdWHs are promising candidates for electronic and optoelectronic applications including transistors, sensors and energy-harvesting devices.^58,67^

**Fig. 7:**
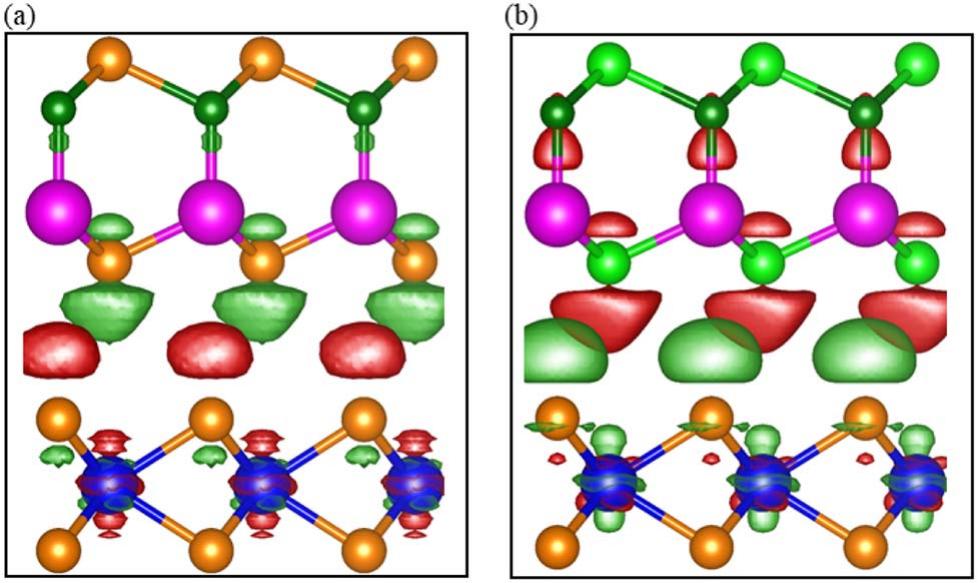
Charge density differences of (a) VS_2_–BGaS_2_ and (b) VS_2_–BGaSe_2_ (see text for details).

The charge transportation is further validated through charge density difference: △*ρ* = *ρ*_(VS_2_–BGaX_2_)_ − *ρ*_(VS_2_)_ − *ρ*_(BGaX_2_)_ and Bader charge analysis, as shown in Fig. 7. In the case of VS_2_–BGaS_2_ MS vdWHs, the charge depletion is absorbed around the BGaS_2_, while charge accumulation around the VS_2_ layer highlights that BGaS_2_ loses and VS_2_ gains electrons, which creates a hole-rich environment in BGaS_2_ and an electron-rich region around VS_2_ layers.^68^ Although , in the case of VS_2_–BGaSe_2_ MS vdWHs, a hole (electron) rich region was established around the VS_2_ (BGaSe_2_) layer. The Bader charge analysis was used to quantitatively evaluate the charge transfer and shows that, in the case of (a) VS_2_–BGaS_2_, 0.003 (0.003) h (e) are transferred between VS_2_ (BGaS_2_), while in the case of (b) VS_2_–BGaSe_2_, 0.0104 (0.0105) h (e) are transferred between VS_2_ (BGaSe_2_). The phenomenon of charge transfer demonstrates a robust interlayer coupling and vdW interactions at the interface of VS_2_ and BGaX_2_ layers within VS_2_–BGaX_2_ MS vdWHs. This charge transport facilitates the formation of a built-in-electric field at the interface, creating a charge separation region and generating an interfacial electric field^66^ that enhances the carrier mobility and increases the concentration of carriers (holes and electrons). Similar experimental results have been confirmed in graphene/GaSe^64^ and graphene/MoS_2_.^65^

**Fig. 8:**
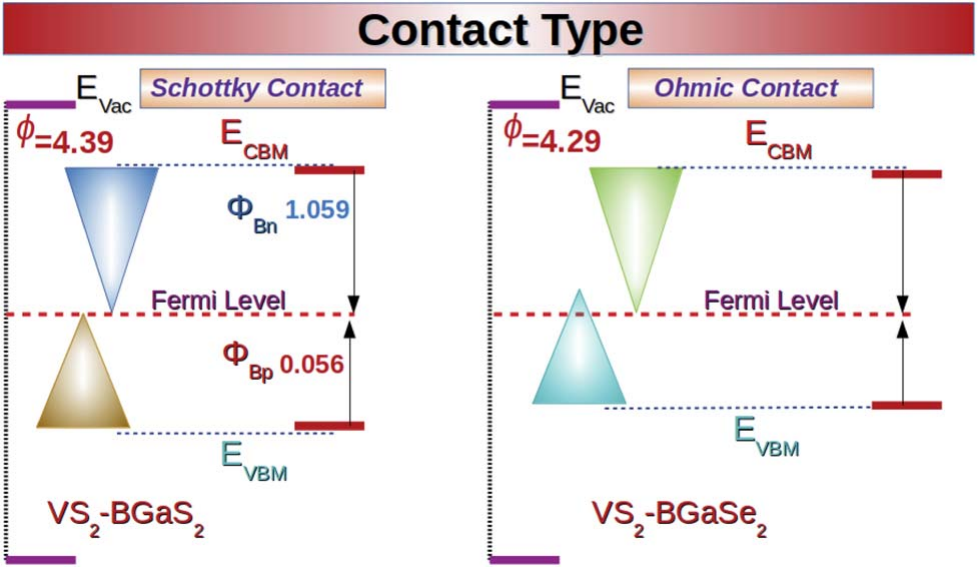
Calculated contact resistance in VS_2_–BGaS_2_ (Schottky) and in VS_2_–BGaSe_2_ (Ohmic) MS vdWHs.

The contact resistance can be tuned through modulation of the Schottky barrier height (SBH) using the electronic structure to regulate the device efficiency.^53,54^ Therefore, it is crucial to understand the barrier height and type at the interface of VS_2_–BGaX_2_ (X = S, Se) MS vdWHs. Fig. 8 shows that a Schottky contact is demonstrated at the interface of the VS_2_–BGaS_2_ MS vdWH,^69^ where *Φ*_Bp_ (0.0564) is found to be lower than *Φ*_Bn_ (1.0594) (see Fig. 8 and computational details), highlighting the formation of a p-type Schottky contact that favors holes conduction over electrons, and plays a significant role in enhancing the performance of phototransistors and photodiodes.^70–72^ In the case of VS_2_–BGaSe_2_ MS contacts, the induced strain and strong electronegativity differences between the VS_2_ and BGaSe_2_ layers cause a shift in the VBM of the semiconducting BGaSe_2_ layer toward the Fermi level, hence, affecting the interlayer coupling and band alignment at the interface. These changes provide adequate control over the contact’s properties and reduce the contact resistance, ultimately leading to the formation of an Ohmic contact. WSe_2_/Ti_2_CF_2_ and WSe_2_/Ti_2_C(OH)_2_ in ref. 73 have already been shown to be Ohmic contacts. The calculated band bending (Δ*ϕ*) for VS_2_–BGaS_2_ vdWHs is −0.223 eV, and for VS_2_–BGaSe_2_ vdWHs is −0.353 eV further confirming a p-type Schottky contact at the interface of VS_2_–BGaX_2_ (X = S, Se) vdWHs.

Unlike conventional TMDC systems, the asymmetric VS_2_–BGaX_2_ (X = S, Se) heterostructures disrupt vertical symmetry due to the differences in the atomic size and electronegativity of X = S, Se, leading to the formation of inequivalent bond lengths across the interface. Therefore, more electrons are transferred from the V atoms to S than Se. This charge localization creates an intrinsic dipole (ID) in the out-of-plane directions, which further alter the transportation of charge at the interface of the VS_2_ and BGaX_2_ layers. The ID is also enhanced due to the weak vdW forces, which promote charge density redistribution at the interface, (as already discussed; see Fig. 6).

Fermi level pinning (FLP), effected by ID at the MS interface, is quantitatively analyzed using the slope parameter *S*:^74^
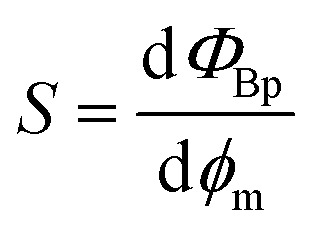
, where *Φ*_Bp_ is the Schottky barrier height (SBH) for a p-type semiconductor and *ϕ*_m_ is the metal work function. Generally, in the Schottky–Mott limit, *S* approaches 1 reflecting no Fermi level pinning (insignificant charge transfer between semiconductor and metal), while *S* approaches 0 for the strongly pinned Fermi level, called the Bardeen limit, where the SBH becomes unaffected by the metal work function. These findings suggest that the FLP profoundly influences the SBH and charge injection, which enhances the overall performance of field-effect transistors (FETs). The calculated *S* parameter for VS_2_–BGaS_2_ is 0.0114, and for VS_2_–BGaSe_2_ vdWHs is 0.056, indicating very low pinning at the interface.^75^

Induced strain in pristine monolayers while fabricating VS_2_–BGaX_2_ MS vdWHs and strong differences in electronegativity also alter the orbitals overlapping, creating a tunneling barrier (TB) at the interface. Lowering the TB can improve carrier mobility, subsequently increasing the current at the contact interface. The TB involves both the tunnel barrier height (*Φ*_TB_ is the potential difference between the vdW gap (*Φ*_gap_) and the potential energy of BGaX_2_ (*Φ*_BGaX_2__)) and the tunnel width (*W*_TB_ indicate the width of the square potential barrier at the interface).^73^ Tuning the Schottky barrier height and minimizing the tunneling barrier width are effective strategies to enhancing charge injection and reducing contact resistance at the MS interface. Therefore, the tunneling barrier probability,^49^*P*_TB_, can be expressed as a comprehensive factor *C* (see computational details). A smaller *C* value is beneficial, as it indicates a higher tunneling barrier probability. In the case of VS_2_–BGaS_2_ and VS_2_–BGaSe_2_ the tunnel barrier height, *Φ*_TB_ (tunnel width, *W*_TB_) is 3.009 eV (3.015 Å) and 2.341 eV (2.73 Å) respectively. For these vdWHs the tunneling barrier probability *P*_TB_ and the comprehensive factor (*C*) are 23.83% (27.361) and 60.82% (17.448). These calculations suggest that VS_2_–BGaSe_2_ exhibits greater charge transfer than in VS_2_–BGaS_2_.

## 4. Conclusion

In this study, we systematically explored the role of interface-induced strain and interlayer coupling in modulating the contact resistance of VS_2_–BGaX_2_ (X = S, Se) vdWHs using DFT calculations. Our findings reveal that these materials exhibit mechanical, thermal and dynamical stability confirmed *via* an energy–strain approach, AIMD simulations, binding energy analysis, and phonon spectrum calculations. The electronic band structure demonstrates that all VS_2_–BGaX_2_ (X = S, Se) MS vdWHs are metal with type-III band alignment, providing a deeper understanding of the interface mechanisms, tunneling characteristics and electronic transport properties across these heterostructures. The work functions of VS_2_–BGaS_2_ and VS_2_–BGaSe_2_ MS vdWHs are 4.399 eV and 4.293 eV, respectively, which are lower than their respective monolayers, modulating charge transfer efficiency. Charge depletion (accumulation) is observed in BGaS_2_ (VS_2_) and VS_2_ (BGaSe_2_) layers confirming the loss (gain) of electrons that creates hole (electron) rich environments. This charge transportation highlights strong interlayer coupling driven by the vdW interactions at the interface of the VS_2_–BGaX_2_ (X = S, Se) MS contact. Importantly, a p-type Schottky contact is identified at the VS_2_–BGaS_2_ interface and an Ohmic contact at the VS_2_–BGaSe_2_ interface with lowering (increasing) of the tunneling probabilities (current), confirming its potential in high-speed nanoelectronic devices. Our findings show that the differences in the contact resistance behavior arise from the impact of induced strain and electronic coupling at the interfaces, enabling tunable functionalities for minimizing power losses and ensuring efficient current injection in transistors and other semiconductor devices due to their built-in potential for charge carriers selectivity. Overall, our results offer valuable insights into the mechanism of contact resistance in 2D MS vdWHs, and serve as a strategic framework for experimentalists to design high-performance solar cells, photodetectors, phototransistors, sensors, and high-speed rectifying devices for next-generation technologies.

## Conflicts of interest

There are no conflicts to declare.

## Supplementary Material

NA-OLF-D5NA00356C-s001

## Data Availability

The data that support the findings of this study are available on request. The data that support the findings of this study in the manuscript and supplementary informations are available on request. See DOI: https://doi.org/10.1039/d5na00356c.
