# Supplementary material for: Role of induced-strain and interlayer coupling in contact resistance of VS2–BGaX2 (X = S, Se) van der Waals heterostructures
Source: Nanoscale Adv. 2025 Jul 28;7(20):6514–24. doi: 10.1039/d5na00356c (PMC12405776; doi:10.1039/d5na00356c)
Supplement: NA-007-D5NA00356C-s001 [file NA-007-D5NA00356C-s001.pdf]

Supplementary Information (SI) for Nanoscale Advances.
This journal is © The Royal Society of Chemistry 2025

Supplementary Informations

Role of induced-strain and interlayer coupling in contact resistance of VS_2-$BGaX_2$ (X=S, Se) van der Waals Heterostructure

Umair Khan[1], Basit Ali[1], Tahani A. Alrebdi[2], M. Bilal[1], M. Shafiq[1], M. Idrees[3], Bin Amin[1*]

[1] *Department of Physics, Abbottabad University of Science & Technology, Abbottabad 22010, Pakistan,* [2] *Department of Physics, College of Science, Princess Nourah bint Abdulrahman University, P.O. Box 84428, Riyadh 11671, Saudi Arabia,*

[3] *School of Chemistry and Chemical Engineering, Shandong University, Jinan, 250100, P. R. China*

* binukhn@gmail.com,+92-333-943-665

I. STRAIN ENGINEERING OF BGAX$_2$ (X= S, SE) AND VS$_2$ MONOLAYERS

We have applied both compressive and tensile strain in the range of 2%, 4%, 6% 8% and 10% on BGaX$_2$ (X= S, Se) and VS$_2$ monolayer to analyzing the nature of their electronic structure. The calculated bandstucture of BGaS$_2$(BGaSe$_2$) and VS$_2$2 monolayer under compressive and tensile strains are illustrated in Figure 1S and Figure 2S, while the top row represent the band structure of BGaS$_2$, second row is for BGaSe$_2$ and thrid row is for VS$_2$ layers. In the compressive strain the bandgap of BGaS$_2$ and BGaSe$_2$ monolayer are gradually decrease and the band nature remains indicate at (K-Γ) points upto 6% compression, while the VS$_2$ remain metallic in nature see Figure 1S. At -8% and -10% compression the transition is occur in the band structure at symmetric point in the Brillouin Zones from (K-Γ) to (M-Γ). The calculated band gap value for BGaS$_2$(BGaSe$_2$) at 2%, 4%, 6%, 8% and 10% compression are 1.298(0.804)eV, 1.148(0.731) eV, 1.03(0.58)eV, 0.798(0.259), 0.366(0.458) eV respectively. For tensile strain's in Figure 2S, the electronic band nature are are remain same like 0% compression and tensile strain the band nature lies on ($_2$) points in the case of BGaS$_2$ and BGaSe$_2$ but the CBM of these monolayers are gradually come closer to the Fermi-level resulting in decrease the bandwidth of these monolayer. Interestingly, at 10% tension the bandgap of BGaSe$_2$ is vanishes the CBM cross the Fermi-level, while the VS$_2$ remain metal similar to unstrain and compressives strain. The calculated bandgap value of BGaS$_2$(BGaSe$_2$) at 2%, 4%, 6% , 8% and 10% tensile strain are 1.31(0.806) eV, 1.291(0.584)eV, 1.281(0.525)eV, 1.059(0.452) eV, and 0.886(0.00)eV respectively.

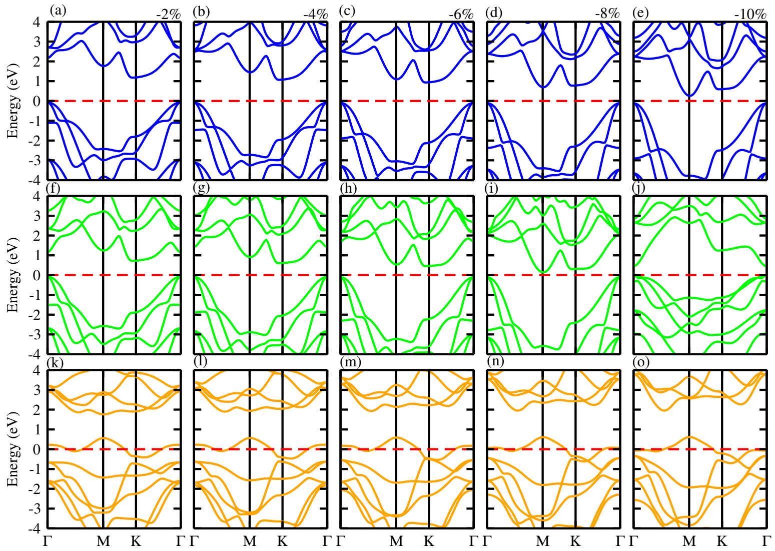

Figure 1S. Electronic band structure of $BGaS_2$(first row), $BGaSe_2$(second row) and VS_2(third row) under tensile strain.

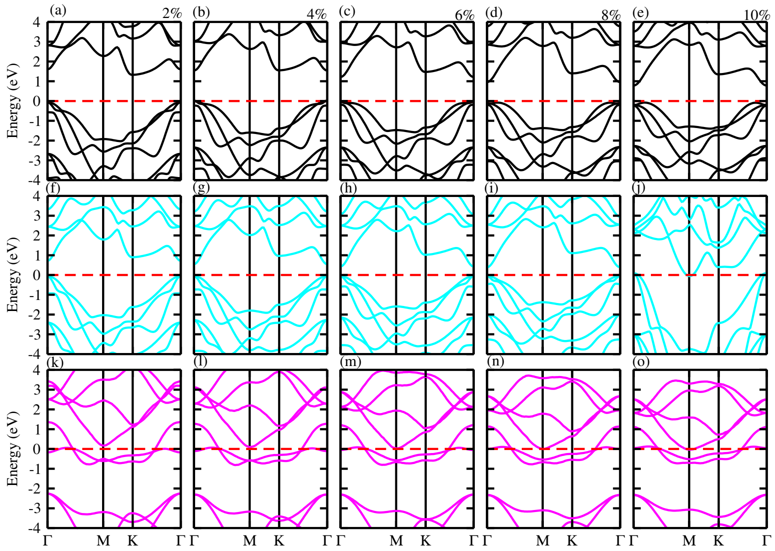

Figure 2S. Electronic band structure of $BGaS_2$(first row), $BGaSe_2$(second row) and VS_2(third row) under compressive strain.

II. ELECTRONIC BAND STRUCTURE OF SIX POSSIBLE STACKING CONFIGURATION OF VS_2-BGAX$_2$ VAN DER WAALS HETEROSTRUCTURES

The difference in the binding energies and the interlayer distance in the Table II of the manuscript show the experimental realizatio of the all six possible stacking configurations of VS_2-BGaX$_2$ (X=S, Se) vdWHs. Although, based on maximum(minimum) binding energy(inter layer distance), we have investigated the most stable configuration in very detail, in the manuscript, while the electronic band structure of the all six stacking configuration are presented in Figure 3S, display energy dispersion along Γ–K–M–Γ. Figure 3S show that, VS_2-BGaX$_2$ in all the six possible configurations ((a)–(f)) are metals with type-III band alignment. See the revised manuscript for detail.

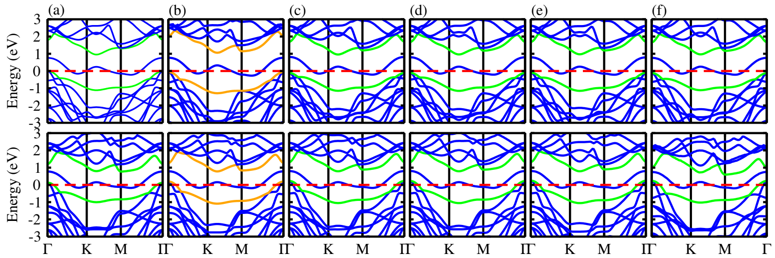

Figure 3. Electronic band structure of six different stacking pattern configurations ((a)–(f)) of VS_2-BGaS$_2$(first row) and VS_2-BGaSe$_2$(second row).

III. ACKNOWLEDGMENT

The authors extend their sincere appreciation to Princess Nourah bint Abdulrahman University Researchers Supporting Project number (PNURSP2025R71), Princess Nourah bint Abdulrahman University, Riyadh, Saudi Arabia
